# The impact of diet and oral hygiene on dental caries among Turkish children: A cross-sectional study

**DOI:** 10.1371/journal.pone.0338081

**Published:** 2025-12-17

**Authors:** Ozge Yesildemir, Melisa Ozay Sekendiz, Duygu Ağagündüz, Ferenc Budán

**Affiliations:** 1 Department of Nutrition and Dietetics, Faculty of Health Sciences, Bursa Uludag University, Bursa, Türkiye; 2 Okan University Dental Hospital, İstanbul, Türkiye; 3 Department of Nutrition and Dietetics, Faculty of Health Sciences, Gazi University, Ankara, Türkiye; 4 Institute of Physiology, Medical School, University of Pécs, Pécs, Hungary; Shahid Beheshti University of Medical Sciences School of Dentistry, IRAN, ISLAMIC REPUBLIC OF

## Abstract

**Background:**

Dental caries remains one of the most prevalent chronic conditions affecting children worldwide, yet they are largely preventable through modifiable factors such as diet and oral hygiene. This study aimed to examine the associations between dietary intake, oral hygiene practices, anthropometric measurements, and dental caries in children.

**Methods:**

This cross-sectional study was conducted in Bursa, Türkiye, between 1 October 2023 and 20 February 2024 with 210 children aged 5–12 years. Data was collected through a structured questionnaire on sociodemographic factors, oral hygiene, and a food frequency questionnaire related to dental health. Body weight and height were measured, and dental examinations were performed by a trained dentist using DMFT/dmft indices.

**Results:**

Mean DMFT and dmft scores were 0.9 ± 1.3 and 3.9 ± 2.8, respectively. Children who brushed their teeth had significantly lower caries scores (*p* < 0.001); brushing twice daily was associated with lower dmft scores (*p* < 0.001). Caries scores did not differ significantly by body mass index (*p* > 0.05). Higher DMFT scores were associated with citrus fruits (*β* = 0.322, *p* < 0.001), white bread (*β* = 0.423, *p* < 0.001), and fruit juice (*β* = 0.050, *p* < 0.05) consumption. Higher dmft scores were linked to chocolate (*β* = 0.286), biscuits, cookies, and cakes (*β* = 0.448), chips (*β* = 0.179), and carbonated soft drinks (*β* = 0.789) (*p* < 0.001).

**Conclusions:**

These results highlight the importance of promoting healthy eating and proper oral hygiene to prevent childhood dental caries and suggest that early oral health status may have broader implications for long-term systemic health, warranting further longitudinal investigation.

## 1. Introduction

The World Dental Federation defines oral health as a multifaceted term that includes the ability to speak, smile, smell, taste, touch, chew, swallow, and convey emotions through facial expressions without pain, discomfort, or disease of the craniofacial complex [1]. Oral health is also an essential indicator of general health status and quality of life because it reflects physiological, social, and psychological attributes [2]. However, oral and dental diseases are currently global public health problems because of their prevalence, treatment costs, and impact on quality of life [3]. Untreated dental caries remains a leading global health issue among children. As of 2023, approximately 17% of children aged 6–9 years worldwide have untreated caries in their primary or permanent teeth [4].

The most common oral and dental diseases include dental caries (tooth decay or dental cavities) and periodontal problems. Dental caries is among the most common chronic diseases in children [5]. It has been reported that 53.8% of children worldwide have decayed permanent teeth, and 46.2% have decayed primary teeth [6]. Türkiye’s Oral and Dental Health Profile Research Report highlighted that the incidence of tooth decay in children aged 5 and 12 years was 64.4% and 46.6%, respectively [7].

Among children, dental caries can lead to embarrassment, anxiety, absenteeism from school, attention problems, learning disorders, early tooth loss, speech disorders, sleep problems, and chewing difficulties [8]. In addition, tooth decay may cause decreased appetite, food intake difficulties, and poor quality of life in children. It can prevent the growth and development of children by negatively affecting their nutrition over time. Owing to the high incidence of dental caries and its undesirable consequences in children, taking comprehensive precautions against tooth decay, a preventable disease, has gained considerable importance in recent decades [9].

Dental caries is a multifactorial disease in which oral hygiene, dietary habits, saliva composition, oral bacterial flora, hypoplasia, and socioeconomic factors affect the formation and progression of caries [10]. However, the most critical risk factors for tooth decay include inadequate dental care and unhealthy eating habits [11]. One way to prevent an increase in cavities is by implementing optimal oral hygiene practices, including various efforts or behaviors to avoid oral and dental diseases and maintain oral health and cleanliness. This encompasses daily brushing and flossing, determining the optimal time for tooth brushing, and seeing a dentist regularly for dental exams and cleaning [12].

Dietary habits play a significant role in the etiology of caries [13]. The type, structure, consumption amount, and frequency of foods and beverages directly influence the integrity of the teeth, saliva composition, oral pH, and microbial activity. This finding highlights the importance of dietary intake in oral and dental diseases, especially tooth decay [5]. Sugars are the most important nutritional factor in the development of dental caries [14]. Approximately 90% of food sugar or starch allows bacteria in plaques to produce acids within 20 minutes after consumption, which leads to mineral loss in the enamel, causing cavities [15]. Conversely, some foods that stimulate saliva secretion, such as hard cheeses, unsweetened yogurt, peanuts, whole-grain foods, and sugar-free chewing gum, have a protective effect against caries. In addition, milk and dairy products may have an anticariogenic effect due to their calcium and phosphorus contents [14]. The presence of calcium in dairy foods stimulates salivary secretion. This also affects the balance between enamel demineralization and remineralization by effectively increasing the calcium concentration in dental plaque, thus constituting a protective factor against dental caries [16]. Recent large-scale international studies have demonstrated that overall dietary habits, including sugar intake, snack frequency, fruit and vegetable consumption, and dairy intake, are significantly associated with dental caries prevalence among children in various countries, including the United States and the Netherlands [17–18]. Therefore, the development of dental caries in children may be reduced by dietary adjustments that consider cariogenic and anticariogenic foods [19].

While the assessment of dietary intake is often not implemented systematically, it is increasingly recognized as an important component of dental preventive strategies, particularly in children [19]. Although many researchers have evaluated the relationship between dietary intake and dental caries among children in Türkiye, they did not use a validated and reliable food frequency questionnaire specifically structured to assess the consumption patterns of food items related to dental caries [20–22]. However, a food frequency questionnaire has recently been developed to evaluate the consumption of food items related to dental caries in Turkish children [19]. To the authors’ knowledge, this study is the first to use this validated and reliable food frequency questionnaire.

Obesity and tooth decay share several common, modifiable factors, such as diet and lifestyle [23]. Nevertheless, whether and how obesity and dental caries are associated is still controversial [24]. Many studies have investigated the association between obesity and cavities; however, existing evidence remains uncertain and inconclusive. While most systematic reviews have shown that overweight or obesity and dental caries in older children (in permanent teeth) are positively associated, the results are less consistent in preschool children (in primary teeth) [24–26]. Therefore, this study aimed to examine relationships among dental caries, oral hygiene practices, dietary intake, and anthropometric measurements in children, using a validated dietary assessment tool specific to caries-related foods. We hypothesize that higher consumption of cariogenic foods (e.g., sugar-sweetened beverages, chocolate, pastries, chips, and rice) and lower consumption of protective foods (e.g., milk, yogurt, cheese, and fruits), along with inadequate oral hygiene practices (e.g., less frequent tooth brushing) and higher anthropometric measurements (e.g., body weight), are positively associated with the prevalence of dental caries in children.

## 2. Materials and methods

### 2.1. Study setting and population

This descriptive and cross-sectional study was conducted with 210 children (57.1% boys, 42.9% girls) aged 5−12 years who applied to a private dental clinic in Bursa, Türkiye, between 1 October 2023 and 20 February 2024. The required sample size was estimated a priori using G*Power software (version 3.0.10), assuming a two-sided alpha level of 0.05 and 80% power, which indicated a minimum of 205 participants. The clinic is located in an urban region and serves families from a wide socioeconomic spectrum. Children aged between 5 and 12 years who agreed to participate in the study were included in the sampling. The sampling method used was convenience sampling, where all eligible children presenting to the clinic during the study period were invited to participate. The exclusion criteria were as follows: (1) having a physical and/or mental disorder affecting oral and dental health; (2) having a known chronic disease influencing nutrition; (3) using regular medication; and (4) using systemic fluoride supplements (such as tablets or drops for caries prevention). Children who received routine topical fluoride applications (e.g., varnish or gel) were not excluded, as these interventions are common in dental practice and have limited long-term systemic effects. In Türkiye, systemic fluoride supplements (such as tablets or drops) are no longer used, and routine fluoride intake is primarily ensured through topical applications, including toothpaste, gels, and varnishes. This study was approved by the Ethics Committee of Bursa Uludag University (Ethics Code: 2023-17/1) on September 19, 2023. All participants provided written informed consent prior to enrollment in the study. This research was conducted ethically in accordance with the World Medical Association Declaration of Helsinki.

### 2.2. Study design

Data on children and their families were collected through face‒to‒face interviews with parents/caregivers via a questionnaire. The questionnaire consisted of questions with general information about the children and their families (child’s gender, age, disease status, parental educational status, profession, income status, etc.), children’s oral hygiene practices (tooth brushing status, frequency, and duration of dental visits, etc.), and a food frequency questionnaire regarding dental health. After the dentist (MO) performed the dental examination of the children, the dietitian (OY) took anthropometric measurements of the children.

### 2.3. Dietary intake

Children’s dietary intake was assessed through a 40-item questionnaire associated with dental health, a food frequency questionnaire developed and validated by Madalı and colleagues in 2023. It consists of cariogenic (biscuits, cookies, cakes, bread, cereal, chips, bananas, dried fruits, jam, honey, etc.) and anticariogenic (milk, yogurt, cheese, apples, etc.) foods [19]. Cariogenic foods cause caries by decreasing the pH of the saliva when it comes into contact with microorganisms in the mouth, and anticariogenic foods protect the tooth surface after acidic foods are consumed [21]. The dietitian (OY) obtained the frequency and amount of food that the children consumed with the help of their parents/caregivers. Each item had seven response categories: never, less than once a month, once a month, 2 times per month, 1–3 times per week, 3–5 times per week, and once a day. These frequency categories were converted into average daily consumption values (e.g., “once a month” = 0.033/day, “1–3 times per week” = 0.29/day). The “Photographic Atlas of Food Portions Sizes” developed for the Turkish population, was used to determine the amounts of food consumed precisely [27]. Portion sizes were multiplied by the numeric daily frequency values to calculate estimated daily intake in grams for each food item, allowing quantitative analysis of dietary intake and its association with dental caries.

### 2.4. Dental examination

Oral and dental examinations of the children were performed by a dentist (MO) under reflector light with the help of a mirror and a probe according to the World Health Organization (WHO) criteria [28]. Dental caries was detected visually with optimal illumination of the oral cavity; no X-rays were used. Standard dental indices (decayed, missing, and filled teeth [DMFT/dmft] or decayed, missing, and filled tooth surfaces [DMFS/dmfs]) were used to measure dental caries in children. The use of both indices aimed at providing a more detailed assessment of caries experience. Decayed, missing, and filled teeth and their surfaces were calculated via the DMFT/S indices for permanent dentition and the dmft/s indices for primary dentition. Based on DMFT/dmft values, the World Health Organization has generated a scale to classify caries severity: these values are classified as very low (<1.2), low (1.2–2.6), moderate (2.7–4.4), high (4.5–6.5), or very high (>6.5) [28]. To assess intra-examiner reliability, 10% of the children were randomly re-examined by the same dentist (MO) one week after the initial examination. The kappa coefficient for DMFT/dmft indices was calculated, and values ranged from 0.85 to 0.92, indicating excellent agreement.

### 2.5. Anthropometric measurements

The body weights and heights of the children were measured by a dietitian (OY). Body weight was measured while the participants wore minimal clothing to the nearest 0.1 kg with a digital scale (Medisana 48435). Height was determined without shoes via a stadiometer (Seca 213). Body mass index (BMI) was calculated and assessed according to z scores via the AnthroPlus program (WHO, Geneva, Switzerland). The children were grouped into four categories according to BMI according to age: underweight, normal, overweight, and obese, with cutoff points of <−1SD, ≥ −1SD– + 1SD, ≥+1SD– + 2SD and ≥+2SD z scores, respectively [29].

### 2.6. Statistical analysis

All the statistical analyses were performed via SPSS Version 23.0 (IBM Inc., Armonk, NY, USA). Numerical variables are expressed as arithmetic means (means) and standard deviations (SDs). Categorical variables are expressed as numbers (n) and percentages (%). The Kolmogorov‒Smirnov test was used to determine if the variables were normally distributed. Independent samples t tests, Mann‒Whitney U tests, one-way ANOVA, or Kruskal‒Wallis tests were applied to determine differences between groups. The Pearson or Spearman correlation coefficient was used to analyze the correlation between the parameters. Multiple linear regression analyses were used to evaluate factors associated with dental indices. Statistical significance was set at p < 0.05.

## 3. Results

The general characteristics of the children and their parents are given in Table 1. Among all the children, 57.1% were boys and 42.9% were girls, with a mean age of 7.5 ± 1.9 years. Most children had normal BMI (57.1%), while 21.4% were underweight and 21.5% overweight. About half of the parents had a primary school education. Most families had middle income (household income equal to household expenses), and none were classified as high income (household income exceeding expenses). Most children brushed their teeth (78.6%), with 54.5% brushing twice daily, and all children used fluoride toothpaste. While brushing duration varied, a majority brushed for 2–3 minutes. Most children (71.4%) had their first dental visit between the ages of 5 and 10 years, usually prompted by complaints (64.3%) (Table 1).

**Table 1:** General characteristics of the children and their parents (n = 210).

Characteristics of children	
Gender, n (%)	
Boy	120 (57.1)
Girl	90 (42.9)
Age (years), mean±SD	7.5 ± 1.9
Anthropometric measurements, mean±SD	
Height (cm)	123.6 ± 14.6
Body weight (kg)	24.6 ± 6.6
Body mass index (kg/m^2^)	15.7 ± 1.5
Body mass index classification, n (%)	
Underweight	45 (21.4)
Normal	120 (57.1)
Overweight	45 (21.5)
**Oral hygiene practices of children**	
Tooth brushing, n (%)	
Brushing	165 (78.6)
Not brushing	45 (21.4)
Frequency of tooth brushing, n (%)	
Once a day	75 (45.5)
Twice a day	90 (54.5)
Duration of tooth brushing, n (%)	
< 2 minutes	60 (36.4)
2-3 minutes	75 (45.5)
> 3 minutes	30 (18.1)
Using fluoride toothpaste, n (%)	
Using	165 (100.0)
Not using	0 (0.0)
Age for first dental visit, n (%)	
0-4 years	60 (28.6)
5-10 years	150 (71.4)
Frequency of dental visits, n (%)	
1-2 times/year	75 (35.7)
Irregular	135 (64.3)
Receiving oral health education, n (%)	
Receiving	75 (35.7)
Not receiving	135 (64.3)
**Dental indices of children**	
Permanent dentition, mean±SD (min-max)	
DMFT	0.9 ± 1.3 (0-4)
DMFS	1.1 ± 1.5 (0-4)
Primary dentition, mean±SD (min-max)	
dmft	3.9 ± 2.8 (1-8)
dmfs	11.0 ± 9.1 (2-28)
**Characteristics of parents**	
Father’s age (years), mean±SD	36.4 ± 3.4
Mother’s age (years), mean±SD	34.4 ± 4.0
Father’s level of education, n (%)	
Primary school	105 (50.0)
High school	60 (28.6)
University	45 (21.4)
Mother’s level of education, n (%)	
Primary school	110 (52.4)
High school	70 (33.4)
University	30 (14.2)
Income status, n (%)	
Low	60 (28.6)
Middle	150 (71.4)

**Fig 1:**
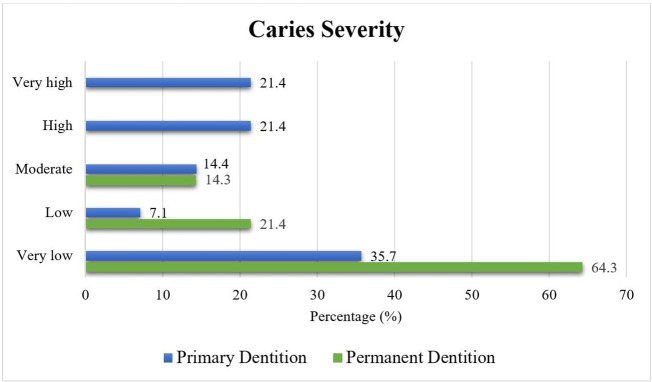
Distribution of children according to the classification of caries severity.

The mean values of the DMFT/S and dmft/s indices in the children were 0.9 ± 1.3, 1.1 ± 1.5, 3.9 ± 2.8, and 11.0 ± 9.1, respectively (Table 1). Most children had very few caries in their permanent dentition (64.3%), while the severity of caries in primary dentition was more evenly distributed, with 42.8% in the low/very low group and 42.8% in the high/very high group (Fig 1).

The mean values of the dental indices associated with oral hygiene practices and the anthropometric measurements of the children are shown in Table 2. The DMFT/S and dmft/s scores of the children who brushed their teeth were lower than those of the children who did not (p < 0.001). Similarly, brushing twice daily was associated with lower dmft/s values compared with brushing once daily (p < 0.001). Early first dental visits and receiving oral health education were both linked to lower caries scores (p < 0.05 and p < 0.001, respectively).

**Table 2:** Comparison of dental indices by oral hygiene practices and anthropometric data of children (n = 210).

	Permanent dentition				Primary dentition			
	DMFT	*p*	DMFS	*p*	dmft	*p*	dmfs	*p*
	Mean±SD		Mean±SD		Mean±SD		Mean±SD	
Tooth brushing								
Brushing	0.5 ± 0.9	**<0.001**	0.7 ± 1.3	**<0.001**	3.0 ± 2.3	**<0.001**	8.1 ± 7.2	**<0.001**
Not brushing	2.3 ± 1.7		2.3 ± 1.7		7.3 ± 1.0		21.7 ± 7.0	
Frequency of tooth brushing								
Once a day	0.4 ± 0.8	0.056	0.8 ± 1.6	0.510	4.4 ± 2.4	**<0.001**	10.8 ± 8.4	**<0.001**
Twice a day	0.7 ± 0.9		0.7 ± 0.9		1.8 ± 1.5		5.8 ± 5.0	
Duration of tooth brushing								
< 2 minutes	1.0 ± 1.0^a^	**<0.001**	1.5 ± 1.7^a^	**<0.001**	3.0 ± 2.1	0.387	6.8 ± 4.0	0.090
2-3 minutes	0.3 ± 0.1^b^		0.2 ± 0.0^b^		3.2 ± 2.9		9.4 ± 9.8	
> 3 minutes	0.0 ± 0.0^b^		0.0 ± 1.0^b^		2.5 ± 0.5		7.5 ± 2.5	
Age for first dental visit								
0-4 years	0.5 ± 0.9	**0.002**	0.5 ± 0.9	**<0.001**	1.0 ± 0.0	**<0.001**	2.5 ± 0.9	**<0.001**
5-10 years	1.1 ± 1.5		1.3 ± 1.7		5.1 ± 2.4		14.4 ± 8.6	
Frequency of dental visits								
1-2 times/year	0.8 ± 1.0	0.300	0.8 ± 1.0	0.056	3.4 ± 2.7	0.059	11.4 ± 8.7	0.634
Irregular	1.0 ± 1.5		1.2 ± 1.8		4.2 ± 2.7		10.8 ± 9.2	
Receiving oral health education								
Receiving	0.8 ± 1.0	0.300	0.8 ± 0.9	0.056	2.0 ± 1.6	**<0.001**	6.6 ± 5.2	**<0.001**
Not receiving	1.0 ± 1.5		1.2 ± 1.7		5.0 ± 2.7		13.4 ± 9.8	
Body mass index classification								
Underweight	0.9 ± 0.1	0.402	0.8 ± 0.1	0.058	5.3 ± 2.1	0.408	15.0 ± 8.3	0.068
Normal	1.6 ± 1.4		1.2 ± 1.6		3.9 ± 2.9		11.6 ± 9.6	
Overweight	1.2 ± 0.9		0.5 ± 0.3		4.7 ± 2.4		8.3 ± 4.8	

The Independent samples t-test and the Mann–Whitney U test were applied for normally and non-normally distributed variables, respectively. The One-way ANOVA and Kruskal–Wallis test were applied for normally and non-normally distributed variables in comparisons among three groups, respectively. The bold values indicate statistical significance (p < 0.05). The groups with the same letters within a column are not significantly different according to pairwise comparisons.

Supplementary Table S1 Table shows the children’s dental indices based on their daily consumption of cariogenic and anticariogenic foods. The DMFT/S values were significantly lower in children who consumed milk, yogurt, cheese, apples, and berries daily (p < 0.05), whereas they were higher in those who consumed ice cream, fruit juice, and cola (p < 0.05). Similarly, children who consumed milk, yogurt, and cheese every day had significantly lower dmft/s scores than those who did not (p < 0.05). Daily consumption of ice cream, citrus fruits, chocolate and chocolate bars, biscuits, cookies, cakes, chips, and fruit juice was associated with higher primary dentition indices (p < 0.001) (Supplementary Table S1 Table).

The correlations between the quantity of food consumed and the dental indices are presented in Table 3. Lower consumption of cheese and apples was significantly associated with higher DMFT scores (p < 0.001). In contrast, higher intake of ice cream, citrus fruits, dried fruits, white bread, sugar in beverages, chocolate and chocolate bars, chips, pastry, spreadable chocolate, fruit juice, and cola was linked to higher DMFT scores (p < 0.001). Additionally, lower consumption of milk, probiotic yogurt, cheese, apples, and berries corresponded to higher dmft scores (p < 0.05), whereas higher intake of ice cream, bananas, dried fruits, white bread, rice, sugar in beverages, sticky candies and bonbons, chocolate and chocolate bars, biscuits, cookies, cakes, chips, honey, spreadable chocolate, fruit juice, and cola was associated with higher dmft scores (p < 0.001) (Table 3).

**Table 3:** Correlation between the quantity of consumption of cariogenic or anticariogenic foods and dental indices.

	Permanent dentition				Primary dentition			
	DMFT		DMFS		dmft		dmfs	
	r	*p*	r	*p*	r	*p*	r	*p*
**Anti-cariogenic Foods**								
Milk (mL)	−0.071	0.308	−0.203	**0.003**	−0.333	**<0.001**	−0.450	**<0.001**
Yogurt (g)	0.092	0.186	−0.025	0.716	−0.062	0.375	−0.023	0.739
Probiotic yogurt (g)	0.117	0.092	0.066	0.344	−0.407	**<0.001**	−0.326	**<0.001**
Cheese (g)	−0.341	**<0.001**	−0.295	**<0.001**	−0.217	**0.002**	0.006	0.931
Apple (g)	−0.280	**<0.001**	−0.151	**0.029**	−0.213	**0.002**	−0.278	**<0.001**
Berries (g)	0.184	0.080	0.117	0.090	−0.218	**0.001**	0.006	0.933
**Cariogenic Foods**								
Ice cream (g)	0.342	**<0.001**	0.232	**<0.001**	0.320	**<0.001**	0.502	**<0.001**
Citrus fruits (g)	0.409	**<0.001**	0.379	**<0.001**	−0.103	0.235	−0.125	0.071
Banana (g)	0.032	0.641	−0.111	0.109	0.344	**<0.001**	0.428	**<0.001**
Dried fruits (g)	0.377	**<0.001**	0.376	**<0.001**	0.255	**<0.001**	0.261	**<0.001**
White bread (g)	0.251	**<0.001**	0.244	**<0.001**	0.181	**<0.001**	0.023	0.744
Rice (g)	0.100	0.150	0.117	0.090	0.414	**<0.001**	0.292	**<0.001**
Sugar in beverages (g)	0.282	**<0.001**	0.156	**0.024**	0.581	**<0.001**	0.591	**<0.001**
Sticky candy, bonbon (g)	−0.070	0.315	−0.011	0.878	0.719	**<0.001**	0.634	**<0.001**
Chocolate, chocolate bars (g)	0.237	**<0.001**	0.207	**0.003**	0.573	**<0.001**	0.564	**<0.001**
Biscuits, cookies, cakes (g)	0.097	0.160	0.094	0.176	0.522	**<0.001**	0.576	**<0.001**
Chips (g)	0.248	**<0.001**	0.224	**0.001**	0.670	**<0.001**	0.580	**<0.001**
Pastry (g)	0.660	**<0.001**	0.645	**<0.001**	0.107	0.121	0.194	**0.005**
Jam (g)	0.097	0.159	0.028	0.684	−0.141	0.051	0.048	0.489
Honey (g)	0.140	0.053	0.052	0.457	0.478	**<0.001**	0.515	**<0.001**
Spreadable chocolate (g)	0.396	**<0.001**	0.313	**<0.001**	0.639	**<0.001**	0.746	**<0.001**
Fruit juice (mL)	0.282	**<0.001**	0.303	**<0.001**	0.304	**<0.001**	0.337	**<0.001**
Cola (mL)	0.553	**<0.001**	0.461	**<0.001**	0.453	**<0.001**	0.576	**<0.001**

Pearson correlation coefficients were calculated for normally distributed variables, while Spearman correlation coefficients were used for non-normally distributed variables. Significant correlations are indicated in bold (p < 0.05). The bold values indicate statistical significance (p < 0.05).

Multiple linear regression analyses were performed to examine the associations between various food groups, demographic characteristics, oral hygiene behaviors, and dental caries indices. The models showed high explanatory power, with adjusted R² values of 0.942 and 0.948 for DMFT and dmft scores, respectively (p < 0.001). Older age was significantly associated with higher DMFT scores (β = 0.397, p < 0.001). Higher maternal and paternal education levels (β = −0.302 and −0.120), higher income (β = −0.753), and more frequent tooth brushing (β = −0.329) were associated with lower DMFT scores (p < 0.001). Among food groups, the consumption of apples did not affect the DMFT score (p > 0.05). Conversely, the consumption of citrus fruits (p < 0.001), white bread (p < 0.001), or fruit juice (p < 0.05) affected the DMFT score. A one-unit increase in citrus fruits, white bread, and fruit juice consumption resulted in 0.322, 0.423, and 0.050 unit increases in the DMFT score, respectively. For dmft scores, older age was related to higher values (β = 0.113, p < 0.001), while higher maternal and parental education (β = −0.100 and −0.134, p < 0.05), higher income (β = −1.162, p < 0.001), and more frequent tooth brushing (β = −0.900, p < 0.001) were linked to lower dmft scores. Higher consumption of chocolate (β = 0.286), biscuits, cookies, and cakes (β = 0.448), chips (β = 0.179), and carbonated soft drinks (β = 0.789) was associated with higher dmft scores (p < 0.001), whereas dairy product intake showed no significant association with dmft values (p > 0.05) (Table 4).

**Table 4:** Multiple linear regression models for various parameters and their associations with dental indices.

	DMFT			
	*β*	95% CI	*p*	Adjusted *R*^2^
Age	0.397	0.227; 0.315	**<0.001**	0.942
Mother’s level of education	−0.302	−0.698; −0.455	**<0.001**	
Father’s level of education	−0.120	−0.315; −0.087	**<0.001**	
Income status	−0.753	−2.441; −2.010	**<0.001**	
Frequency of tooth brushing	−0.329	−0.013; −0.009	**<0.001**	
Apple	0.016	−0.001; 0.001	0.458	
Citrus fruits	0.322	0.425; 0.689	**<0.001**	
White bread	0.423	0.032; 0.045	**<0.001**	
Fruit juice	0.050	0.000; 0.001	**0.031**	
	**dmft**			
	***β***	**95% CI**	***p***	**Adjusted *R*** ^**2**^
Age	0.113	0.071; 0.249	**<0.001**	0.948
Mother’s level of education	−0.100	−0.003; −0.001	**0.004**	
Father’s level of education	−0.134	−0.882; −0.047	**0.029**	
Income status	−1.162	−4.703; −3.610	**<0.001**	
Frequency of tooth brushing	−0.900	−3.495; −2.944	**<0.001**	
Dairy products	−0.017	−0.266; 0.148	0.574	
Chocolate	0.286	0.034; 0.047	**<0.001**	
Biscuits, cookies, cakes	0.448	0.022; 0.026	**<0.001**	
Chips	0.179	0.009; 0.019	**<0.001**	
Carbonated soft drinks	0.789	2.813; 3.419	**<0.001**	

Multiple linear regression analyses were performed to examine associations between food groups, demographic characteristics, oral hygiene behaviors, and dental indices (DMFT and dmft). β refers to the regression coefficient representing the change in dental indices associated with each variable. Bold values indicate statistical significance (p < 0.05). Variable values: Mother’s and Father’s level of education (Primary school = 1, High school = 2, University = 3); Income status (Low = 0, Middle = 1); Frequency of tooth brushing (Not brushing = 1, Once a day = 2, Twice a day = 3).

## 4. Discussion

This study assessed children aged 5–12 years and demonstrated that oral hygiene practices and dietary intake significantly affect children’s development of dental caries. Tooth brushing frequency and duration, the timing of the first dental visit, and receiving oral health education were all associated with dental index scores. However, no significant difference in caries incidence was observed between BMI groups. Although there were negative relationships between dairy products such as milk, probiotic yogurt, and cheese and some dental index scores, these relationships were not significant in the regression models. In contrast, citrus fruits, white bread, chocolate, biscuits, cookies, cakes, chips, fruit juice, and carbonated soft drinks were associated with increased risk of dental caries [30].

The DMFT/S and dmft/s indices are widely used in epidemiological studies to assess and monitor dental health globally [31]. One of the WHO oral health goals for children under 12 years of age is to maintain a mean DMFT/dmft value less than 1.5 [32]. In our study, this target was achieved for permanent teeth, whereas primary teeth exhibited higher caries scores, indicating a greater burden in the deciduous dentition. It should be noted that the data were collected from a dental clinic and therefore may not be representative of the general population.

Brushing habits had a clear impact on caries prevalence. Children who brushed twice daily for at least two minutes had significantly lower dmft/s and DMFT/S scores, confirming the importance of adequate oral hygiene—especially for vulnerable primary teeth [33,34]. As all participants used fluoride toothpaste, the effect of fluoride could not be isolated. Contrary to expectations, no significant association was observed between recent dental visits and caries scores. The late timing of first visits (mostly between ages 5–10) and the low rate of regular check-ups likely limited their preventive benefits [35,36].

Socioeconomic characteristics were also important predictors of oral health. Lower parental education and household income were significantly associated with higher caries rates, suggesting a protective effect of higher socioeconomic status. These results align with prior studies indicating that socioeconomic disadvantage contributes to increased caries prevalence through lower health literacy, limited access to care, and suboptimal oral hygiene behaviors [37–39]. Moreover, nearly two-thirds of the children had never received formal oral health education, emphasizing the need for early, school-based preventive interventions [37–39].

Unraveling the relationship between obesity and dental caries remains complex, as systematic reviews have yielded inconsistent results—some reporting a positive association, others finding no significant link, and some even suggesting an inverse relationship [26,40,41]. In line with previous findings in Turkish children [5], no significant association was observed between BMI and caries experience in this study. This may reflect the influence of contextual factors such as dietary patterns, oral hygiene practices, and healthcare access. For instance, stronger associations between obesity and caries have been observed in high-income countries, where consumption of sugar-rich foods and sedentary lifestyles are more prevalent [42]. It is also possible that oral hygiene behaviors and socioeconomic factors exert a more direct influence on caries risk than BMI alone [41].

Teeth are in daily contact with a variety of foods, and the type and composition of these foods play a key role in caries development [43]. In this study, dairy products—particularly milk, probiotic yogurt, and cheese—were initially associated with lower caries scores, in line with previous findings that emphasize the benefits of calcium, casein, and phosphate in buffering plaque acidity and promoting enamel remineralization [21,44,45]. Probiotic yogurts, in particular, may offer additional protection by supporting microbial balance and reducing *Streptococcus mutans* colonization [46]. However, when included in multiple linear regression models alongside other dietary and sociodemographic factors, these associations were not statistically significant. This suggests that the apparent protective effect of dairy products may be modest relative to stronger cariogenic influences, such as sugar-sweetened beverages or pastries, or may be attenuated by variability in product type and composition. Flavored or sweetened dairy items, for example, could counteract the potential benefits due to added sugars [47,48]. Therefore, while dairy consumption may contribute to caries prevention, its independent effect is limited when considering the broader dietary and demographic context.

Among fruits, apples showed a negative association with caries indices in correlation analyses, likely due to their fibrous texture, which stimulates salivary flow and facilitates mechanical cleaning [49–51]. However, their natural acids may contribute to enamel erosion depending on frequency and timing of consumption [52], and they may influence oral microbiota composition in complex ways [53]. In regression models adjusted for other dietary and sociodemographic variables, the protective effect of apples was not statistically significant, indicating that their independent contribution to caries reduction is modest.

In contrast to apples, certain fruits were linked to increased caries risk. Bananas, for instance, are starchy and easily fermentable, making them a potential substrate for acidogenic bacteria [21]. Their low pH may further promote enamel demineralization in children [54]. Citrus fruits, rich in organic acids like citric and malic acid, can lower oral pH and cause enamel erosion when consumed frequently, especially as snacks [55]. While some studies report that consuming citrus fruits with meals may mitigate these effects [56,57], the evidence remains inconclusive. Additionally, several epidemiological studies indicate that high consumption of citrus fruits is associated with increased caries experience—for example, in citrus-producing farm workers compared to non-fruit growing control groups [58]. The high acidity can facilitate demineralization and when combined with the natural sugars present in these fruits, it may promote cariogenic bacterial activity [59]. It should be noted that epidemiological evidence specifically linking whole citrus fruit consumption to dental caries is limited, and further studies are needed to clarify this relationship. Similarly, dried fruits, due to their stickiness and high sugar content, pose a caries risk, although more robust research is needed to confirm their long-term effects [60].

The association between high sugar intake and dental caries is well-established. Our study further supports this link, finding a significant association between frequent sugary food and beverage consumption and increased caries in children, underscoring the critical role of dietary habits in oral health. Evidence shows that maintaining free sugar intake below 10% of total energy significantly reduces caries risk in children [61]. Sugary and carbonated beverages create an acidic environment by lowering plaque pH and eroding enamel directly [21,62]. Although dark chocolate contains polyphenols with antimicrobial potential that may protect against caries [63], the high sugar content in most commercial chocolate products often outweighs these benefits, increasing cariogenic risk [64]. Interestingly, some studies report no clear link between sugar and caries when oral hygiene is adequate [65], indicating that consumption patterns and brushing behavior strongly modify this association.

The role of starchy foods in dental caries remains a topic of debate. In this study, starchy foods included white bread, rice, biscuits, cookies, cakes, chips, and pastry, as recorded in dietary intake data. While starches themselves are not inherently cariogenic, refined and sticky starchy foods can be broken down into fermentable sugars and adhere to tooth surfaces, providing substrates for acidogenic bacteria [61]. However, the literature presents mixed findings. Several studies report associations between high intake of starchy foods and caries development in children [11,20–22,61,62], whereas others suggest no significant link when sugar consumption and oral hygiene practices are adequately controlled [66]. In our study, frequent consumption of processed starchy foods, particularly biscuits, chips, and pastries, was associated with higher caries prevalence, highlighting that both food type and oral hygiene behavior influence starch-related cariogenicity.

### 4.1. Limitations

This study has several limitations that should be considered when interpreting the results. First, the use of a convenience sampling method at a single private dental clinic in an urban area of Bursa may limit the generalizability of the findings to broader populations, including rural or socioeconomically different groups. Second, the cross-sectional design prevents any causal inferences regarding the relationships between dietary intake, oral hygiene practices, and dental caries. Third, dental examinations relied solely on visual inspection using DMFT/dmft indices, without radiographic confirmation or international caries detection and assessment system (ICDAS) scoring. Consequently, early or non-cavitated lesions may have been underestimated, limiting the sensitivity of caries detection. Fourth, dietary intake was assessed through a parent-reported food frequency questionnaire, which is subject to recall bias and may not fully capture actual consumption patterns. To minimize this risk, we used a previously validated questionnaire; however, the inherent limitations of self-reported dietary data remain. Fifth, anthropometric measurements and BMI categorization, while standardized, do not provide information about body composition or fat distribution, which may influence health outcomes differently. Sixth, the wide age range of 5–12 years included in the study, although controlled for in regression models, may still introduce variability due to differences in dentition stages and caries risk profiles. Finally, important potential confounding factors such as socioeconomic status, oral microbiome composition, and fluoride exposure beyond toothpaste use were not comprehensively controlled or analyzed. Future research should address these limitations through longitudinal, community-based studies with larger and more diverse samples, comprehensive clinical and biochemical assessments, and more robust dietary evaluation methods, including ICDAS or other sensitive caries detection tools, to better understand the complex interactions affecting childhood dental health.

## 5. Conclusions

In conclusion, this study demonstrated that dental caries in childhood is significantly associated with oral hygiene practices and dietary intake. Brushing teeth at least twice daily for approximately two minutes, timely dental visits, and receiving oral health education were all positively linked to better dental outcomes. Additionally, the influence of certain cariogenic and anticariogenic foods on caries development was observed. These findings suggest that both oral hygiene habits and dietary patterns contribute to the risk of dental caries. While acknowledging the limitations inherent in this cross-sectional design, the results underscore the importance of further investigating the complex relationships between oral hygiene behaviors, diet, and dental health in children. Future research employing longitudinal or interventional designs is needed to clarify these associations. Importantly, oral health education should be integrated with nutritional guidance to effectively promote comprehensive preventive strategies.

## Supporting information

S1 File Dataset. (XLSX)

S2 File Inclusivity-in-global-research-questionnaire. (DOCX)

S1 Table Dental indices of children by the daily consumption of cariogenic and anticariogenic foods. (DOCX)
