# Supplementary material for: The impact of diet and oral hygiene on dental caries among Turkish children: A cross-sectional study
Source: PLoS One. 2025 Dec 17;20(12):e0338081. doi: 10.1371/journal.pone.0338081 (PMC12711038; doi:10.1371/journal.pone.0338081)
Supplement: S1 Table — (DOCX) [file pone.0338081.s003.docx]

**Supplementary Table S1.** Dental indices of children by the daily consumption of cariogenic and anticariogenic foods.

|  | **Permanent Dentition** | | | | **Primary Dentition** | | | |
| --- | --- | --- | --- | --- | --- | --- | --- | --- |
|  | **DMFT** | ***p*** | **DMFS** | ***p*** | **dmft** | ***p*** | **dmfs** | ***p*** |
|  | **Mean±SD** |  | **Mean±SD** |  | **Mean±SD** |  | **Mean±SD** |  |
| **Anti-cariogenic Foods**  Milk |  |  |  |  |  |  |  |  |
| Consuming (n:60) | 0.5±0.9 | **0.004** | 0.5±0.9 | **<0.001** | 1.0±0.0 | **<0.001** | 2.5±0.9 | **<0.001** |
| Not consuming (n:150) | 1.1±1.5 |  | 1.3±1.7 |  | 5.1±2.4 |  | 14.4±8.6 |  |
| Yogurt |  |  |  |  |  |  |  |  |
| Consuming (n:60) | 0.0±0.0 | **<0.001** | 0.0±0.0 | **<0.001** | 3.7±2.7 | **<0.001** | 10.3±8.7 | **0.009** |
| Not consuming (n:150) | 1.1±1.4 |  | 1.3±1.6 |  | 5.5±2.5 |  | 15.0±10.2 |  |
| Cheese |  |  |  |  |  |  |  |  |
| Consuming (n:60) | 0.5±0.9 | **0.003** | 0.5±0.9 | **<0.001** | 1.3±0.4 | **<0.001** | 4.0±3.5 | **<0.001** |
| Not consuming (n:150) | 1.1±1.5 |  | 1.3±1.7 |  | 5.0±2.6 |  | 13.8±9.1 |  |
| Apple |  |  |  |  |  |  |  |  |
| Consuming (n:15) | 0.0±0.0 | **0.005** | 0.0±0.0 | **0.005** | 3.0±0.0 | 0.179 | 5.0±0.0 | **0.007** |
| Not consuming (n:195) | 1.0±1.4 |  | 1.2±1.6 |  | 4.0±2.9 |  | 11.5±9.2 |  |
| Berries |  |  |  |  |  |  |  |  |
| Consuming (n:12) | 0.0±0.0 | **0.013** | 0.0±0.0 | **0.013** | 3.1±1.8 | 0.328 | 8.2±6.1 | 0.265 |
| Not consuming (n:198) | 1.0±1.4 |  | 1.1±1.6 |  | 4.0±2.8 |  | 11.1±9.1 |  |
| **Cariogenic Foods** |  |  |  |  |  |  |  |  |
| Ice cream |  |  |  |  |  |  |  |  |
| Consuming (n:15) | 1.0±1.4 | **0.005** | 1.2±1.6 | **0.005** | 8.0±0.0 | **<0.001** | 26.0±0.0 | **<0.001** |
| Not consuming (n:195) | 0.0±0.0 |  | 0.0±0.0 |  | 3.6±2.6 |  | 9.8±8.3 |  |
| Citrus fruits |  |  |  |  |  |  |  |  |
| Consuming (n:15) | 0.8±1.0 | 0.700 | 1.6±12.0 | 0.168 | 6.4±2.0 | **<0.001** | 19.2±8.6 | **<0.001** |
| Not consuming (n:195) | 0.0±1.4 |  | 1.0±1.5 |  | 3.7±2.7 |  | 10.4±8.8 |  |
| Banana |  |  |  |  |  |  |  |  |
| Consuming (n:46) | 0.8±1.4 | 0.477 | 1.0±1.7 | 0.643 | 4.0±2.8 | 0.751 | 10.8±9.3 | 0.869 |
| Not consuming (n:164) | 1.0±1.3 |  | 1.1±1.5 |  | 3.9±2.8 |  | 11.1±9.0 |  |
| Sugar in tea, coffee or beverages |  |  |  |  |  |  |  |  |
| Consuming (n:46) | 1.1±1.4 | 0.200 | 1.2±1.4 | 0.688 | 4.3±2.7 | 0.223 | 10.0±7.2 | 0.398 |
| Not consuming (n:164) | 0.9±1.3 |  | 1.0±1.6 |  | 3.8±2.8 |  | 11.3±9.5 |  |
| Sticky candy, bonbon |  |  |  |  |  |  |  |  |
| Consuming (n:59) | 0.5±0.9 | 0.054 | 1.0±1.8 | 0.749 | 5.2±1.9 | **<0.001** | 12.9±7.6 | 0.060 |
| Not consuming (n:151) | 1.1±1.4 |  | 1.1±1.4 |  | 3.4±2.9 |  | 10.2±9.5 |  |
| Chocolate, chocolate bars |  |  |  |  |  |  |  |  |
| Consuming (n:42) | 1.0±1.5 | 0.471 | 1.1±1.6 | 0.989 | 6.6±0.9 | **<0.001** | 15.7±5.9 | **<0.001** |
| Not consuming (n:168) | 0.9±1.3 |  | 1.1±1.4 |  | 3.3±2.7 |  | 9.8±9.3 |  |
| Biscuits, cookies, cakes |  |  |  |  |  |  |  |  |
| Consuming (n:26) | 1.1±1.4 | 0.689 | 0.8±1.2 | 0.853 | 8.0±0.0 | **<0.001** | 25.4±0.5 | **<0.001** |
| Not consuming (n:184) | 1.2±1.6 |  | 0.9±1.5 |  | 3.4±2.5 |  | 8.9±7.7 |  |
| Chips |  |  |  |  |  |  |  |  |
| Consuming (n:17) | 0.5±1.3 | 0.141 | 0.5±1.3 | 0.093 | 8.0±0.0 | **<0.001** | 25.4±1.0 | **<0.001** |
| Not consuming (n:193) | 1.0±1.3 |  | 1.1±1.5 |  | 3.6±2.6 |  | 9.7±8.3 |  |
| Spreadable chocolate |  |  |  |  |  |  |  |  |
| Consuming (n:60) | 0.8±1.3 | 0.222 | 0.8±1.3 | 0.055 | 5.2±2.6 | **<0.001** | 12.8±8.2 | 0.076 |
| Not consuming (n:150) | 1.0±1.3 |  | 1.2±1.6 |  | 3.4±2.7 |  | 10.3±9.3 |  |
| Fruit juice |  |  |  |  |  |  |  |  |
| Consuming (n:11) | 4.0±0.0 | **<0.001** | 4.0±0.0 | **<0.001** | 8.0±0.0 | **<0.001** | 28.0±0.0 | **<0.001** |
| Not consuming (n:199) | 0.8±1.2 |  | 0.9±1.4 |  | 3.7±2.7 |  | 10.0±8.3 |  |
| Cola |  |  |  |  |  |  |  |  |
| Consuming (n:15) | 1.0±1.4 | **0.005** | 1.2±1.6 | **0.005** | 6.0±0.0 | **0.002** | 12.0±0.0 | 0.658 |
| Not consuming (n:195) | 0.0±0.0 |  | 0.0±0.0 |  | 3.8±2.8 |  | 10.9±9.4 |  |

The Independent samples t-test and the Mann–Whitney U test were applied for normally and non-normally distributed variables, respectively. The bold values indicate statistical significance (p<0.05).
